# The spectrum of genetic variants and phenotypic features of Southeast Asian patients with Noonan syndrome

**DOI:** 10.1002/mgg3.581

**Published:** 2019-02-19

**Authors:** Ai‐Ling Koh, Ee‐Shien Tan, Maggie S. Brett, Angeline H. M. Lai, Saumya Shekhar Jamuar, Ivy Ng, Ene‐Choo Tan

**Affiliations:** ^1^ Department of Paediatrics KK Women's & Children's Hospital Singapore; ^2^ Genetics Service, Department of Paediatrics KK Women's & Children's Hospital Singapore; ^3^ Paediatrics Academic Clinical Programme SingHealth Duke‐NUS Graduate Medical School Singapore; ^4^ Research Laboratory KK Women's & Children's Hospital Singapore

**Keywords:** genetic testing, molecular diagnosis, Noonan syndrome, RASopathies

## Abstract

**Background:**

Noonan syndrome (NS) is an autosomal dominant disorder that belongs to a group of developmental disorders called RASopathies with overlapping features and multiple causative genes. The aim of the study was to identify mutations underlying this disorder in patients from Southeast Asia and characterize their clinical presentations.

**Methods:**

Patients were identified from the hospital's Genetics clinics after assessment by attending clinical geneticists. A targeted gene panel was used for next‐generation sequencing on genomic DNA extracted from the blood samples of 17 patients.

**Results:**

Heterozygous missense variants were identified in 13 patients: eight were in *PTPN11*, three in *SOS1*, and one each in *RIT1* and *KRAS*. All are known variants that have been reported in patients with NS. Of the 13 patients with identified variants, 10 had short stature, the most common feature for NS. Four of the eight patients with *PTPN11* variants had atrial septal defect. Only two had pulmonary stenosis which is reported to be common for *PTPN11* mutation carriers. Another two had hypertrophic cardiomyopathy, a feature which is negatively associated with *PTPN11 *mutations.

**Conclusions:**

Our study provides the mutation and phenotypic spectrum of NS from a new population group. The molecular testing yield of 76% is similar to other studies and shows that the targeted panel approach is useful for identifying genetic mutations in NS which has multiple causative genes. The molecular basis for the phenotypes of the remaining patients remains unknown and would need to be uncovered via sequencing of additional genes or other investigative methods.

## 1 INTRODUCTION

Noonan syndrome (NS, OMIM 163950) is an autosomal dominant disorder with variable expressivity. It is one of the more common Mendelian disorders with the reported frequency of one in 1,000–2,500 live births (Nora et al., 1974). There has been no recent update on the prevalence observed, and also no country‐ or population‐specific data.

Noonan syndrome is caused by germline mutations in genes encoding components belonging to the Ras and mitogen‐activated protein kinase (MAPK) signaling pathway that interacts with extracellular growth factors to regulate cell proliferation, differentiation, and senescence. Although somatic mutations of Ras/MAPK pathway genes are found in many cancers, germline mutations are behind a group of developmental disorders whose presentations include craniofacial dysmorphism, short stature, congenital heart disease, and skeletal abnormalities. The disorders are collectively called RASopathies, among which NS is the most common. RASopathies also include Noonan syndrome with multiple lentigines (NSML) which was previously known as LEOPARD syndrome (OMIM 151100), Costello syndrome (OMIM 218040), cardiofaciocutaneous (CFC) syndrome (OMIM 115150), other Noonan‐like syndrome (OMIM 607721), Legius syndrome (OMIM 611431), and neurofibromatosis type 1 (OMIM 162200). As these syndromes have overlapping clinical features with variable expressivity and some manifestations are also age‐dependent, establishing the correct diagnosis can be challenging.

The variable clinical phenotype behind NS and Noonan‐like disorders is partly due to genetic heterogeneity, where mutations in multiple genes can result in different phenotypic expressions. To date, mutations in *PTPN11 *(OMIM 176876), *SOS1 *(OMIM 182530), *RAF1 *(OMIM 164760), *KRAS *(OMIM 190070)*,* *NRAS *(OMIM 164790), *BRAF *(OMIM 164757)*, RIT1 *(OMIM 609591)*, SOS2 *(OMIM 601247)*, *and *LZTR1 *(OMIM 600574) have been identified in patients with NS, with *PTPN11* accounting for 50%–60% of identified mutations (Aoki & Matsubara, 2013; El Bouchikhi et al., 2016; Rauen, 2013; Tafazoli et al., 2017). For Costello syndrome, mutations in *HRAS* (OMIM 190020) have been identified in 83%–100% of patients and is the only gene definitely associated with the syndrome thus far (Tartaglia et al., 2010). *PTPN11*, *RAF1,* *BRAF, *and *MAP2K1* (OMIM 176872) are mutated for NSML; while *BRAF, KRAS*, *MAP2K1*, and *MAP2K2* (OMIM 601263) are causative genes for CFC syndrome (Pandit et al., 2007; Roberts et al., 2007; Tartaglia et al., 2010).

Some of the identified genes are only associated with one disorder, examples are *HRAS* for Costello syndrome; and *SHOC2 *(OMIM 602775)*, RRAS *(OMIM 165090), and *CBL* (OMIM 165360) for Noonan‐like syndrome (El Bouchikhi et al., 2016; Martinelli et al., 2010). On the other hand, other genes have mutations identified in more than one related disorders, such as *PTPN11 *for both NS and NSML (Pandit et al., 2007; Roberts et al., 2007; Tartaglia et al., 2010), and *KRAS* for NS and CFC (Aoki & Matsubara, 2013), *MAP2K1 *and *SOS1* for CFC and NS (Kouz et al., 2016). For these genes, the resulting disorder would usually depend on whether the specific disease‐causing variant results in gain or loss of function of the encoded protein.

As mutations in different genes have been found in patients with the different disorders, identifying the causative gene mutation would help to differentiate Noonan from Noonan‐like syndromes and other RASopathies. Since different disorders have different prognosis, a molecular confirmation of the clinical diagnosis will result in more targeted medical management to improve the patient's long‐term outcome and quality of life. For example, specific cardiac follow‐up is recommended for hypertrophic cardiomyopathy (HCM) in NS and Costello syndrome (Calcagni et al., 2018). On the other hand, patients with syndromes that have higher risk for malignancy (such as Costello syndrome) require disease‐specific tumor surveillance.

The spectrum of mutations for NS has been reported in European and American populations, and also several countries in Western and Northern Asia. However, the mutation spectrum has not been characterized in patients from Singapore. In this study, we utilized next‐generation sequencing to identify the genetic mutations and evaluate the phenotypic features in our NS patients on genetic follow‐up in Singapore, where >95% of the country's population are of Chinese, Malay, or Indian descent.

## 2 MATERIALS AND METHODS

### 2.1 Ethical compliance and enrollment of subjects

The study was protocol was reviewed and approved by the SingHealth Centralized Institutional Review Board, Singapore. A written informed consent was obtained from all subjects’ parents. Patients with clinical suspicion of NS from the Genetics clinics in KK Women's and Children's Hospital were assessed by the attending clinical geneticists, using the criteria developed by van der Burgt et al. (1994) as a guide.

### 2.2 DNA sequencing

Genomic DNA was extracted from venous blood and sequenced using a custom‐designed targeted panel that included all exons and exon‐intron junctions of *PTPN11, NF1, BRAF, KRAS, NRAS, HRAS, SOS1, RAF1, SHOC2, CBL, RIT, MAP2K1, MAP2K2, SPRED1,* and* RASA1*. The DNA samples were prepared for sequencing using the HaloPlex^TM ^Target Enrichment System (Agilent Technologies, Santa Clara, CA) and sequenced on the MiSeq System (Illumina, San Diego, CA). Data were processed using the MiSeq Reporter pipeline and annotated using wANNOVAR (Chang & Wang, 2012). Sequence variants were checked against those available in dbSNP, 1,000 Genomes, ExAC, ClinVar, and Human Genome Mutation Database. Variants not previously reported in population databases or not previously classified as pathogenic were evaluated using SIFT and Polyphen predictions. All identified variants were confirmed via Sanger sequencing. Targeted Sanger sequencing of parental samples for variants identified in the patients was used to establish inheritance. Information on frequencies in population databases, previous reports in patients with similar phenotypes, and inheritance status were used to classify the pathogenicity of the variants according to the guidelines by ClinGen's RASopathy Expert Panel (Gelb et al., 2018).

## 3 RESULTS

### 3.1 Study subjects

Seventeen unrelated patients were enrolled into the study from July 2014 to November 2015. The demographic characteristics of the patients are presented in Table 1. Nine patients were already on follow‐up with our hospital before study commencement, the remaining eight were recruited during their first consultation with the Genetics Service clinics. The median age at clinical assessment was 3 years old.

**Table 1:** Demographic and clinical characteristics of all patients (*n* = 17) at the time of enrollment with their disease‐causing variants

Patient No.	1	2	3	4	5	6	7	8	9
Ethnic group	Malay	Indonesian	Indian	Malay	Chinese	Chinese	Malay	Chinese	Malay
Gender	Male	Male	Male	Female	Male	Female	Male	Male	Male
Age at recruitment	1 month	11 years	10 months	2 years	7 years	4 months	15 years	7 years	6 years
Gene	*PTPN11*	*PTPN11*	*PTPN11*	*PTPN11*	*PTPN11*	*PTPN11*	*PTPN11*	*PTPN11*	*SOS1*
Nucleotide change	c.188A>G	c.188A>G	c.236A>G	c.417G>C	c.922A>G	c.922A>G	c.1472C>A	c.1510A>G	c.806T>C
Exon	3	3	3	4	8	8	13	13	6
Amino acid change	p.Y63C	p.Y63C	p.Q79R	p.E139D	p.N308D	p.N308D	p.P491H	p.M504V	p.M269T
Inheritance	Unknown	De novo	De novo	Unknown	De novo	Unknown	Unknown	Paternal	De novo
Cardiac condition	ASD, PS	ASD	ASD, PS	HCM	HCM	Normal	Normal	ASD, VSD	Normal
Short stature	+	+	−	+	+	−	+	+	−
Pectus deformity	−	+	+	−	−	−	−	−	+
Shield chest	−	−	−	−	−	+	−	−	−
Cubitus valgus	−	−	−	−	−	−	−	−	−
Scoliosis	−	−	−	−	−	−	+	−	−
Cryptorchidism	+	−	+	N/A	+	N/A	+	+	−
Renal anomaly	−	−	−	−	+	−	−	+	−
Astigmatism	+	+	+	−	−	−	−	−	−
Amblyopia	−	−	+	−	−	−	−	+	−
Learning difficulty	−	−	Mild	Mild	Mild	−	−	Mild	−

Patient No.	10	11	12	13	14	15	16	17
Ethnic group	Chinese	Malay	Indian	Chinese	Malay	Chinese	Chinese	Malay
Gender	Male	Male	Female	Female	Female	Male	Female	Male
Age at recruitment	3 years	7 months	11 years	9 months	4 years	11 days	2 years	3 years
Gene	*SOS1*	*SOS1*	*RIT1*	*KRAS*	−	−	−	−
Nucleotide change	c.1300G>C	c.1300G>C	c.284G>C	c.178G>C				
Exon	10	10	5	2	−	−	−	−
Amino acid change	p.G434R	p.G434R	p.G95A	p.G60R				
Inheritance	De novo	Maternal	De novo	De novo	−	−	−	−
Cardiac condition	−	PS	PS	PS	−	Unknown	−	PS, VSD, HCM
Short stature	+	+	+	+	+	−	−	+
Pectus deformity	+	+	+	−	+	−	−	+
Shield chest	+	−	−	−	−	−	−	−
Cubitus valgus	−	+	+	−	−	−	−	−
Scoliosis	−	−	−	−	−	−	−	−
Cryptorchidism	−	+	N/A	N/A	N/A	−	−	+
Renal anomaly	−	−	−	+	−	−	−	−
Astigmatism	−	−	−	−	−	−	−	−
Amblyopia	−	−	−	−	−			−
Learning difficulty	−	−	Mild	Moderate‐severe	−	Unknown	Mild	Moderate‐severe

Note

Reference transcripts: *PTPN11: *NM_002834.4*, SOS1*: NM_005633.*3, RIT1:* NM_006912.5 *KRAS*: NM_004985.4.

ASD: atrial septal defect; HCM: hypertrophic cardiomyopathy; N/A: not applicable; PS: pulmonary stenosis; VSD: ventricular septal defect.

### 3.2 Clinical evaluation

Complete data on all 17 patients wer only available for eight clinical features (Table 1), of which the most common was short stature (12/17 [70.6%]), followed by pectus deformity (8/17 [47.1%]). For clinical features with incomplete data, the most prevalent was cardiac abnormality (10/16) with pulmonary stenosis (PS) being the most common subtype (6/10), followed by atrial septal defect with four affected patients and HCM with three affected. Eight out of 16 patients had learning difficulty, of which six were mild (studying in mainstream schools but requiring educational intervention such as additional coaching). Only two patients had moderate to severe learning difficulty (requiring special education). One patient was not assessed as he was too young at the time of enrollment and he did not return for follow‐up sessions. The remaining eight had normal intelligence.

### 3.3 Resequencing of target genes

The mean coverage for target bases across the 14 genes was 162x. Heterozygous missense variants were identified in 13 patients: eight were in *PTPN11*, three in *SOS1*, and one each for *RIT1* and *KRAS*. The list of identified variants and clinical phenotypes of individual patients are shown in Table 1.

Seven of the identified variants were de novo, while two were inherited. The inheritance of the identified variants for the remaining four subjects (all *PTPN11*) was unknown as parental samples were not available. For the two variants that were inherited, the father of patient no. 8 with the *PTPN11 *M504V (NM_002834.4:c.1510A>G) variant had short stature with typical facial features of NS, while the mother of patient no. 11 with the *SOS1* G434R (NM_005633.3:c.1300G>A) variant had short stature, short neck, mild hypertelorism, coarse facial features, and cubitus valgus of NS. One of the patients with a de novo variant in *SOS1* also had a paternally inherited *NF1* variant (NM_000267.3:c.3484A>G, p.(Met1162Val)) which is of unknown clinical significance. The father had three café au lait spots >15 mm with right‐sided axillary freckling but no other signs of NF1. The child had café au lait spots on his lower back but no freckling. Both the patient and his father did not fulfill the clinical criteria for NF1.

### 3.4 Genotype–phenotype correlations

The number and percentage of patients carrying disease‐causing variants in each of the four mutated genes and their specific phenotypic feature are shown in Table 2. The most common phenotypic features are facial dysmorphism, (low‐set ears, ocular hypertelorism), short stature, and cardiac defects. The most frequently observed cardiac condition was PS found in five patients. In the eight patients with *PTPN11* mutation, two had PS and another two had HCM diagnosed at age 2 and 7 years respectively.

**Table 2:** Details of phenotypic features in patients with identified mutations (*n* = 13)

Phenotypic feature	No. of patients with the condition/total with mutations in gene				No. of affected/total	%
	*PTPN11*	*SOS1*	*RIT1*	*KRAS*		
Cardiac condition						
ASD	4/8	—	—	—	4/13	30.8
PS	2/8	1/3	1/1	1/1	5/13	38.5
HCM	2/8	—	—	—	2/13	15.4
VSD	1/8	—	—	—	1/13	7.7
Normal	2/8	2/3	—	—	4/13	30.8
Short stature (<3rd centile)	6/8	2/3	1/1	1/1	10/13	76.9
Low‐set ears	8/8	3/3	—	—	11/13	84.6
Ocular hypertelorism	3/8	1/3	1/1	1/1	6/13	46.2
Pectus deformity	2/8	3/3	1/1	—	6/13	46.2
Shield chest	1/8	1/3	—	—	2/13	15.4
Cubitus valgus	—	1/3	1/1	—	2/13	15.6
Scoliosis	1/8	—	—	—	1/13	7.7
Cryptorchidism	5/6	1/3	N/A	N/A	6/9	66.7
Renal abnormalities	2/8	—	—	1/1	3/13	23.1
Astigmatism	3/8	—	—	—	3/13	23.1
Amblyopia	2/8	—	—	—	2/13	15.4
Learning difficulties						
None	4/8	3/3	—	—	7/13	53.8
Mild	4/8	—	1/1	—	5/13	38.5
Moderate‐severe	—	—	—	1/1	1/13	7.7

Note

ASD: atrial septal defect; HCM: hypertrophic cardiomyopathy; PS: pulmonary stenosis; VSD: ventricular septal defect.

Three of the four patients without any identified disease‐causing variant fulfilled the diagnostic criteria for NS. The remaining patient (no. 15 in Table 2) had lower limb lymphedema and was seen by the geneticist when he was 11 days old. He was negative for other clinical features at that time. As he was lost to follow‐up after the second medical review at the age of 3 months, it was uncertain whether additional clinical feature had since developed.

## 4 DISCUSSION

This study identified disease‐causing variants in 13 out of 17 patients with clinical suspicion of NS, similar to other reports where genetic mutations were found in 60%–70% of patients tested. The gene with the largest number of mutations was *PTPN11*. It is also the main gene that accounts for more than half of identified mutations in other studies (El Bouchikhi et al., 2016; Tartaglia et al., 2010). For our eight patients with identified *PTPN11* disease‐causing variants in this study*, *five of them carried three (Y63C, Q79R, and N308D) of the four most common mutations for NS (Aoki & Matsubara, 2013). Seven of the eight disease‐causing variants identified in this study were localized in exons 3, 8, and 13, domains identified as hot‐spots for de novo mutations (El Bouchikhi et al., 2016; Narayanan et al., 2017). Three are in the N‐amino terminal src‐homology 2 domain (N‐SH2) encoded by exons 1–3 or the phosphotyrosine phosphatase encoded by exons 8–13. Only the variant found in patient no. 4 is in exon 4 which encodes the C‐amino terminal src‐homology 2 domain (C‐SH2) (El Bouchikhi et al., 2016).

Three patients had disease‐causing variants in *SOS1*, the second most mutated gene in NS (El Bouchikhi et al., 2016). Two of them had the recurrent G434R mutation reported by multiple studies, while the remaining patient had another known mutation (Kiel & Serrano, 2014; Zenker et al., 2007a). The only disease‐causing *RIT1 *variant found in this study had also been reported (Aoki et al., 2013; Bertola et al., 2014). One patient in our study had a *KRAS* mutation which had been previously reported in patients with CFC syndrome (Niihori et al., 2006; Zenker et al., 2007b).

Of the 13 patients with identified disease‐causing variants, 10 (76.9%) had short stature, with height of less than third centile based on the growth chart for the local population. No adjustment for parental height could be made as data on parental height was not available. The prevalence of short stature from our study was comparable to that previously reported (Kruszka et al., 2017; Romano et al., 2010; Zenker, 2011).

The next most common feature was cardiac abnormalities found in 9 out of 13 patients with identified disease‐causing variants. In addition, patient no. 17 for whom no mutation was identified had three cardiac conditions PS, VSD and HCM are all defined in Table 1. The high proportion of cardiac abnormalities is not surprising as it is a key feature of NS. This syndrome is also the most common syndromic cause of congenital cardiac conditions after trisomy 21 (Marino et al., 1999). However, our prevalence of specific cardiac phenotypes stratified by causative genes appeared to be slightly different from other studies. While the 50% proportion for atrial septal defect for patients with *PTPN11* mutations was in line with reported findings, only two of eight patients had PS. The prevalence of this clinical feature in *PTPN11* mutation carriers had been reported to be 60% (Sznajer et al., 2007), 63% (Cizmarova et al., 2016), 65% (Papadopoulou et al., 2012), 71% (Tartaglia et al., 2002), and 88% (Zenker et al., 2004). For Korean patients, PS was reported in about 46%–50% of patients with *PTPN11* mutations (Ko et al., 2008; Lee et al., 2011). On the other hand, the same number of our patients (two) had HCM, frequently associated with *RAF1* mutations and rare in *PTPN11*‐associated NS (Pandit et al., 2007; Roberts et al., 2007; Tartaglia et al., 2010). As the number of patients was small in our study, analysis with larger cohorts of patients would be necessary to establish if the prevalence of PS is indeed lower for NS patients with *PTPN11* mutations in Asian populations.

Intelligence has been reported to be normal for most patients with NS, with intellectual impairment affecting only about 20% (Allanson, 2007; Pierpont et al., 2009). Seven of our 13 patients with identified disease‐causing variants had normal intelligence; the remaining six had some learning difficulty. All three patients with *SOS1* disease‐causing variants had normal intelligence, consistent with other reports for this gene (Pierpont et al., 2009; Tartaglia et al., 2007; Zenker et al., 2007a). Four out of eight patients with *PTPN11* disease‐causing variants had mild learning difficulty. While *PTPN11* N308D is associated with lower IQ in one study (Cesarini et al., 2009), one of our two patients with this disease‐causing variant had normal intelligence while the other had only mild learning difficulty. The only one with moderate to severe learning difficulty among the 13 patients had a *KRAS* variant, which is not surprising as it has been reported that learning difficulty is more common in patients with *KRAS* mutations (Schubbert et al., 2006; Zenker et al., 2007b). Moderate to severe cognitive impairment is generally more common in CFC patients and can be used to distinguish CFC from NS (Zenker et al., 2007b). However, Patient no. 13 had typical NS phenotypic features and lacked the skin and hair abnormalities commonly seen in CFC syndrome patients. She also had hydrocephalus requiring ventriculoperitoneal shunt, swallowing dysfunction post fundoplication and gastrostomy, sagittal synostosis postsurgical correction, severe obstructive sleep apnea requiring nocturnal noninvasive continuous positive airway pressure ventilation, and congenital hypoplasia of kidney, which could be present in both NS and CFC syndrome patients. Her case demonstrated the overlapping phenotype of these two syndromes in a patient with *KRAS* mutation.

To our knowledge, this is the first genetic study of NS in a Southeast Asian population. While the spectrum of disease‐causing variants in our patients appeared to be similar to populations elsewhere, indicating that there are no population‐specific hotspots; the clinical manifestations seemed to be heterogeneous. Our two pairs of patients with identical disease‐causing variants did not have similar clinical presentations with each other or with what had been reported in other studies for carriers of this variant. Although the four patients who did not have any mutation identified had fewer clinical features, they might have mutations in genes that were not in the panel.

## CONFLICT OF INTEREST

None declared.
